# Signaling Crosstalk Mechanisms That May Fine-Tune Pathogen-Responsive NFκB

**DOI:** 10.3389/fimmu.2019.00433

**Published:** 2019-07-02

**Authors:** Adewunmi Adelaja, Alexander Hoffmann

**Affiliations:** ^1^UCLA-Caltech Medical Scientist Training Program, Department of Microbiology, Immunology, and Molecular Genetics, David Geffen School of Medicine, Molecular Biology Institute, University of California, Los Angeles, Los Angeles, CA, United States; ^2^Department of Microbiology, Immunology, and Molecular Genetics, Institute for Quantitative and Computational Biosciences, University of California, Los Angeles, Los Angeles, CA, United States

**Keywords:** NFκB, PAMPs (pathogen-associated molecular patterns), interferon-beta (IFNβ), signaling crosstalk, immunoproteasome, TRIF, A20 (TNFAIP3), IκBs

## Abstract

Precise control of inflammatory gene expression is critical for effective host defense without excessive tissue damage. The principal regulator of inflammatory gene expression is nuclear factor kappa B (NFκB), a transcription factor. Nuclear NFκB activity is controlled by IκB proteins, whose stimulus-responsive degradation and re-synthesis provide for transient or dynamic regulation. The IκB-NFκB signaling module receives input signals from a variety of pathogen sensors, such as toll-like receptors (TLRs). The molecular components and mechanisms of NFκB signaling are well-understood and have been reviewed elsewhere in detail. Here we review the molecular mechanisms that mediate cross-regulation of TLR-IκB-NFκB signal transduction by signaling pathways that do not activate NFκB themselves, such as interferon signaling pathways. We distinguish between potential regulatory crosstalk mechanisms that (i) occur proximal to TLRs and thus may have stimulus-specific effects, (ii) affect the core IκB-NFκB signaling module to modulate NFκB activation in response to several stimuli. We review some well-documented examples of molecular crosstalk mechanisms and indicate other potential mechanisms whose physiological roles require further study.

## Introduction

**Figure 1:**
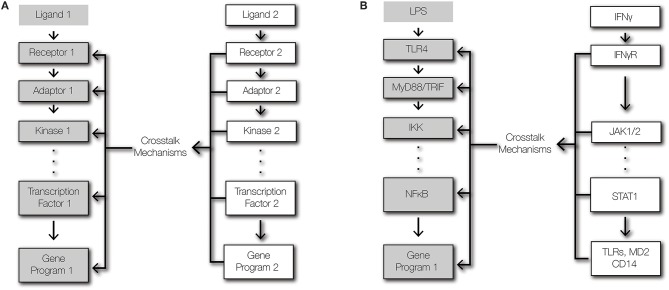
Signaling and crosstalk. **(A)** Regulatory crosstalk is defined here as the signal transduction within a pathway being altered by a second pathway that affects the abundances or functions of signaling components. **(B)** Schematic of signaling crosstalk from IFNγ signaling to TLR4-NFκB signaling.

**Figure 2:**
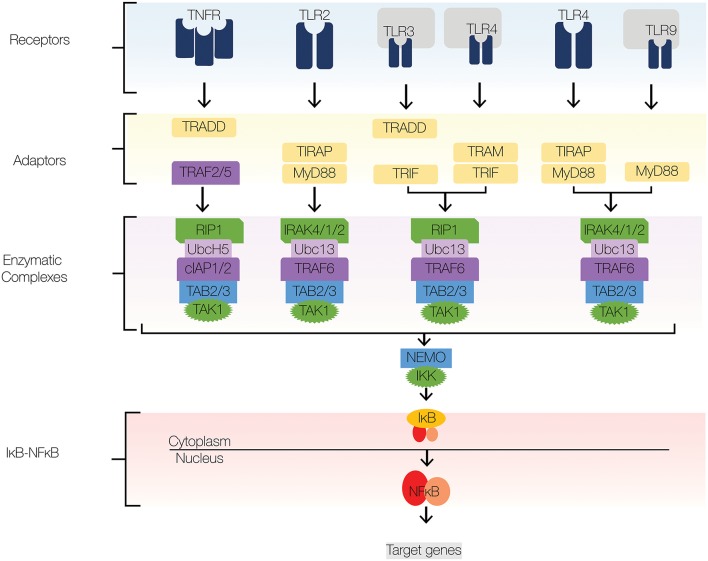
NFκB signaling pathway. The major signaling components of the NFκB signaling pathway include receptors, adaptors, enzymatic complexes, and the IκB-NFκB complex. Upon ligand recognition, cognate receptors engage adaptor proteins that recruit kinases and ubiquitin ligases to the signaling complex. TLR signaling employs adaptor proteins MyD88 and TRIF; both of which contain TIR domains. Sorting adaptor proteins such as TIRAP and TRAM facilitate MyD88 and TRIF association with the signaling complex. MyD88 engages an enzymatic complex that includes IRAK4, IRAK1, IRAK2, TRAF6, Ubc13, TAB2/3, and TAK1. TRIF engages a similar enzymatic complex, which includes RIP1 instead IRAK4,1,2. The enzymatic complexes facilitate the recruitment and activation of IKKβ, which induces the degradation of IκBs and subsequent nuclear translocation of NFκB. Navy blue, TLRs; yellow, adaptors; green, kinases; dark purple, E3 ligases; light purple, E2 conjugases.

NFκB signaling mediates inflammatory and innate immune responses; the signaling components that comprise the core signaling pathway are well-understood and have been amply reviewed, for example by Mitchell et al. (1), Leifer and Medvedev (2), Pandey et al. (3), and Hayden and Ghosh (4). Here, therefore, is only a brief summary. Of 15 possible NFκB dimers, the predominant mediator of NFκB inflammatory gene expression is the ubiquitous RelA:p50 heterodimer (1). At rest, inhibitors of κB (IκB)s sequester RelA:p50 in the cytoplasm by masking its DNA binding region and nuclear localization signal (5–7). In response to stimuli, IκBs are phosphorylated by IκB kinase (IKK), which triggers their ubiquitination and proteolysis (8, 9). Then, RelA:p50 translocates from the cytoplasm to the nucleus, where it binds and activates promoters and enhancers of target genes, such as *nfkbia*, which codes for IκBα (10, 11). Since IκBα synthesis is induced by RelA:p50, a tightly coupled negative feedback loop emerges that regulates NFκB activity in a highly dynamic and stimulus-specific fashion (11–13). To tune NFκB signaling, crosstalk mechanisms regulate signal transduction from TLRs to IκBs to NFκB (Figure 1). We describe crosstalk mechanism at four levels: receptors, adaptors, enzymatic complexes, and the IκB-NFκB signaling module (Figure 2). Here, we focus on a few well-established crosstalk mechanisms, and mention others that deserve further study.

To ensure effective host defense against pathogens and to maintain tissue integrity, immune cells must integrate multiple signals to produce appropriate responses (14). Cells of the innate immune system are equipped with pattern recognition-receptors (PRRs) that detect pathogen-derived molecules, such as lipopolysaccharides and dsRNA (3). Once activated, PRRs initiate series of intracellular biochemical events that converge on transcription factors that regulate powerful inflammatory gene expression programs (15). To tune inflammatory responses, pathways that do not trigger inflammatory responses themselves may modulate signal transduction from PRRs to transcription factors through crosstalk mechanisms (Figure 1). Crosstalk allows cells to shape the inflammatory response to the context of their microenvironment and history (16). Crosstalk between two signaling pathways may emerge due shared signaling components, direct interactions between pathway-specific components, and regulation of the expression level of a pathway-specific component by the other pathway (1, 17). Since toll-like receptors (TLRs) are the best characterized PRRs, they provide the most salient examples of crosstalk at the receptor module. Key determinants of tissue microenvironments are type I and II interferons (IFNs), which do not activate NFκB, but regulate NFκB-dependent gene expression (18–21). As such, this review focuses on the cross-regulation of the TLR-NFκB signaling axis by type I and II IFNs.

Whereas, IFNγ is the only type II IFN, the type I IFN family consists of multiple forms of IFNα and IFNβ (22, 23). Type I IFNs ligate interferon-α receptors (IFNAR), which leads to the activation of Janus-activated kinase-1 (JAK1), tyrosine kinase 2 (Tyk2), and IFN-stimulated gene factor 3 (ISGF3) complex, which consists of signal transducer and activator of transcription 1 (STAT1), STAT2, and IFN-regulatory factor (IRF)-9 (23). IFNγ ligates IFNγ-receptor (IFNGR), which leads to the activation of JAK1 and JAK2 and the subsequent STAT1 phosphorylation and homodimerization (22).

## Receptor Modules

### Receptor Abundance and Localization

**Figure 3:**
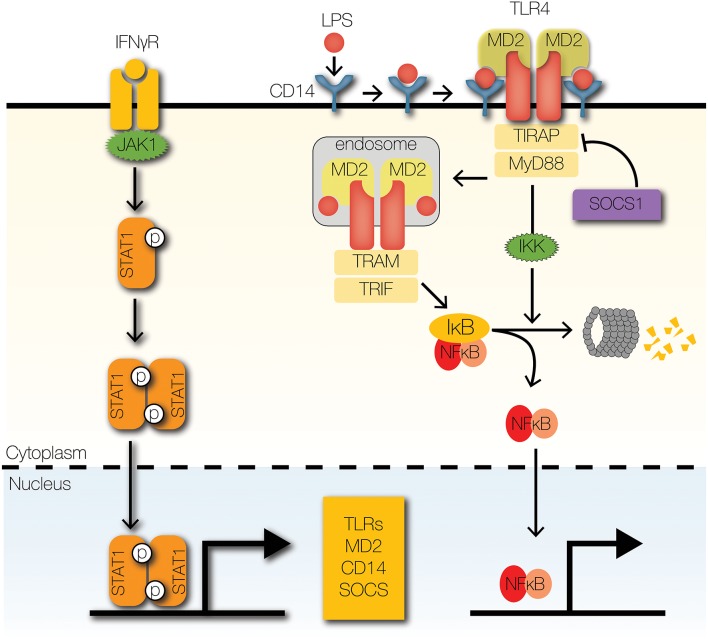
Signaling crosstalk at receptors and adaptors. IFNγ receptor activation leads to the phosphorylation and nuclear translocation of STAT1 homodimers. STAT1 upregulates the expression of several signaling components of the TLR signaling pathway, such asTLRs and co-receptors MD2 and CD14. SOCS1, a STAT1-inducible negative regulator of STAT1 signaling, promotes the degradation of TIRAP by facilitating K48-ubiquitin-mediated proteolysis.

IFNγ is a well-described crosstalk mediator that enhances NFκB signaling (Figure 3) (20). By upregulating the expression of TLRs, IFNγ enhances the detection of pathogen-associated molecular patterns (PAMPs) by TLRs in different cellular compartments. At the plasma membrane, TLR2 and TLR4 recognize microbial cell wall components, such as lipopolysaccharides and lipoproteins (24). Similarly, endosomal TLRs, such as TLR3 and TLR9, recognize double stranded RNA and CpG oligonucleotides (24). IFNγ upregulates TLR2, TLR3, TLR4, and TLR9 at the mRNA and protein levels (25–30). Similarly, the inflammatory cytokine, tumor necrosis factor (TNF) upregulates the mRNA expression of TLR2 (31). The significance of TNF-induced and IFNγ-induced upregulation of TLR abundance on NFκB signaling dynamics is unknown. In addition to recognizing PAMPs, TLRs recognize host-derived molecules, such as extracellular matrix proteins, heat-shock proteins, nucleic acids, and high mobility group box 1 (32–37). Whereas, high TLR abundance facilitates detection of pathogens and mobilization host defenses, it may also increase susceptibility to autoimmune diseases and sepsis (24).

### Accessory Protein Abundance

In addition to upregulating TLR expression, IFNγ also upregulates expression of TLR accessory proteins (Figure 3), such as myeloid differentiation factor 2 (MD2) and CD14 (29, 38, 39). Both accessory proteins facilitate the binding of lipopolysaccharide (LPS) to TLR4, in part by regulating localization of TLR4 (40–42). In fact, MD2 is necessary for localization of TLR4 to the plasma membrane, where it can bind LPS and transduce signals to downstream components (41, 43). After activation, TLR4 undergoes dynamin-mediated endocytosis into endosomes, where it continues transmitting signals (44). In the absence of CD14, endocytosis of TLR4 and subsequent signal transmission are attenuated. Further, CD14 and MD2 promote the association of endosomal TLR4 to downstream adaptors, which are critical for signal transduction (41, 42). Although CD14 is primarily associated with TLR4-mediated signaling, it also facilitates TLR2, TLR3, and TLR9 signaling (45–47). Interestingly, accessory proteins may contribute to inflammation in Alzheimer's disease (AD) and atherosclerosis (48). CD36, a scavenger receptor, recognizes amyloid β and oxidized LDL, which contribute to pathogenesis of AD and atherosclerosis, respectively (48). CD36 forms a heterotrimeric complex with TLR4 and TLR6 to induce production of inflammatory mediators (48). Further, IFNγ-activated macrophages significantly upregulate the expression CD36 in disease models of atherosclerosis (49).

### Signaling Adapters

While IFNγ upregulates the expression of TLRs and accessory proteins that promote inflammatory responses, it also upregulates negative feedback regulators to maintain homeostasis (Figure 3). To enable negative feedback, IFNγ, TNF, and type I IFNs induce the expression of a family of E3 ubiquitin ligases, aptly named suppressors of cytokine signaling (SOCS) (18, 25, 50). SOCS1 was reported as a negative regulator of TLR4 signaling that is essential for the formation of endotoxin tolerance (51). The putative mechanism by which SOCS1 inhibits TLR signaling is through ubiquitin-mediated degradation of TIR domain containing adaptor (TIRAP), which recruits myeloid differentiation primary response gene 88 (MyD88) to TLR2 and TLR4 by mitigating the effects of electrostatic repulsion (52). The significance of SOCS1 is evident from the fact that SOCS1 deficiency causes neonatal lethality in mice due to overwhelming inflammation (53). However, loss of IFNγ rescues *socs1*^−/−^ mice, which suggests that the primary role of SOCS1 is to restrain IFNγ-dependent inflammation and pathology.

Since TLRs do not possess the catalytic activity to activate NFκB directly, they engage adaptors such as MyD88 and TIR-domain-containing adapter-inducing interferon-β (TRIF) to propagate signals downstream (54, 55). The expression of MyD88 may be controlled by IFNγ, since *myd88* mRNA is IFNγ-inducible (25). Furthermore, MyD88 degradation may also be regulated by the anti-inflammatory cytokine, transforming growth factor (TGF)β, through Smad6-dependent recruitment of Smad ubiquitin regulatory factor (Smurf) 1/2 E3 ubiquitin ligases (56). However, the physiological significance of these crosstalk mechanisms remains to be fully elucidated.

## Enzymatic Complexes

**Figure 4:**
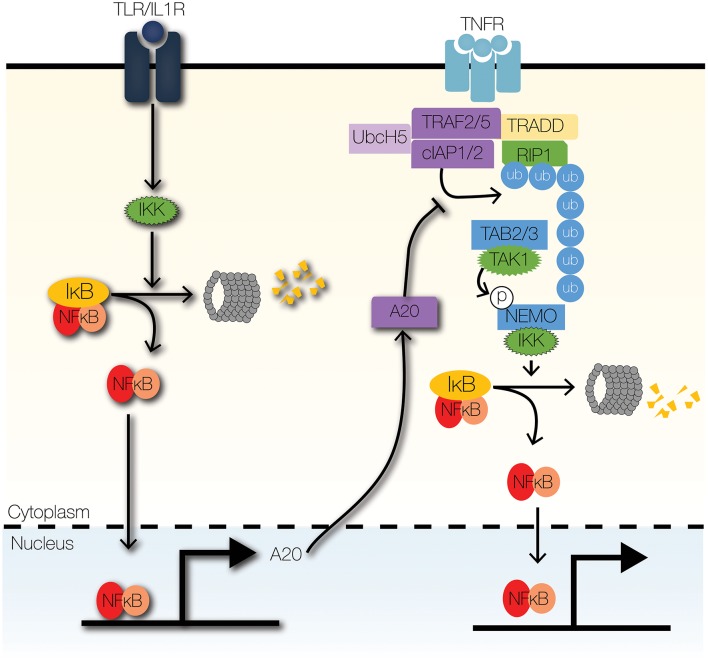
Signaling crosstalk at enzymatic complexes. TLR signaling can modulate TNF signaling through the actions of A20, a ubiquitin-editing enzyme. A20 inhibits the recruitment of IKK to the TNFR signaling complex by inhibiting K63-linked ubiquitination of RIP1. Further, A20 increases the degradation of RIP1 by facilitating K48-linked ubiquitination of RIP1.

Signal transduction from TLRs to NFκB involves recruitment of several enzymes to the TLR signaling complex (3). The recruited kinases and ubiquitin ligases allow for signal amplification while providing pathway specificity (13, 57). The enzymes upstream of the IKK signaling complex provide multiple avenues and nodes for signal integration and crosstalk (57–59). Both the catalytic activity and abundance of these enzymes can be subject to cross-regulation (Figure 4). After engaging TLRs, MyD88 forms an oligomeric complex with IL1R-associated kinases (IRAK) called the Myddosome (60). Formation of the Myddosome complex brings IRAK4 dimers and IRAK1/2 dimers into close proximity for efficient signal transduction (61). In response to IFNγ stimulation, immune cells upregulate the expression of IRAKs and MyD88 (25, 29, 62). In contrast, TNF stimulation upregulates the expression of negative regulators of TLR signaling, such as IRAK-M (63). The expression of IRAK-M in macrophages abrogates signaling downstream of IRAKs, inhibits TLR-induced NFκB activation, and mediates endotoxin tolerance (64). As limiting components in TLR signal transduction, MyD88, and IRAKs form critical junctures for regulatory control of inflammatory responses (60, 65). During endotoxin tolerance, the abundance of IRAKs and the association of TLRs with MyD88 are reduced (62). Therefore, crosstalk at this module can serve a dual purpose: priming and tolerance.

Similar to TNF receptor 1 (TNFR1), TRIF engages the adaptor protein tumor necrosis TNFR1-associated death domain protein (TRADD) and the kinase receptor-interacting protein (RIP)1 (66, 67). NFκB activation through TRIF-RIP1 signaling is dependent on Pellino-1, which is an E3 ubiquitin ligase that is essential for the formation of ubiquitin scaffold on RIP1 (68); however, the E3 ubiquitin ligase activity of Pellino-1 may be dispensable for TRIF-dependent activity (69). Whereas, loss of Pellino-1 expression abolishes TRIF-dependent RIP1 ubiquitination, loss of Pellino-1 E3 ubiquitin ligase activity does not affect RIP1 ubiquitination (68, 69). Although the inducible expression of Pellino-1 mRNA (*Peli1*) is dependent on IFN-regulatory factor 3 (IRF3), evidence suggests *Peli1* is also a target gene of ISGF3, which is induced by type I IFNs (70). Whether type I IFNs enhance TRIF-NFκB in a Pellino-1-dependent manner is unknown. Since the loss of Pellino-1 confers resistance to septic shock in response to TLR3 and TLR4 activation, it is possible that type I IFNs cross-regulate TRIF-NFκB through Pellino-1 to regulate septic shock (68). However, direct evidence is lacking.

The primary E3 ubiquitin ligase that transduces signals from MyD88 to IKK is TRAF6 (71–73). Downstream of IRAKs, TRAF6 facilitates the formation of K63-linked ubiquitin scaffold and the recruitment of IKK to the TLR signaling complex (73). TLR-NFκB signaling is regulated by ubiquitin editing enzymes, such as A20 and cylindromatosis (CYLD) (74, 75). We will focus the next section on A20 though it is not IFN-controlled but provides important signaling crosstalk (Figure 4).

A20 is a highly inducible NFκB target gene that attenuates cytokine- and pathogen-mediated inflammatory signaling (76, 77). Loss of A20 is lethal, due to excessive inflammation, cachexia, and organ failure (78, 79). Furthermore, dysregulated A20 signaling contributes to the pathogenesis of atherosclerosis and rheumatoid arthritis (80–82). A20 is an essential negative feedback regulator and terminator of TLR signaling (77). It edits ubiquitin tags on TRAF6 and RIP1 (75, 83). A20 removes K63-linked ubiquitin chains from RIP1 and may add K48-linked ubiquitin chains to target RIPK1 for proteasomal degradation (75). Additionally, A20 disrupts the interactions between TRAF6 and E2 ubiquitin conjugating enzymes, Ubc13 and UbcH5; A20 also enhances proteasomal degradation of Ubc13 and UbcH5c, by catalyzing the deposition of K48-linked ubiquitin chains (83). By mediating signaling crosstalk between TNFR and TLR/IL1R signaling pathways, A20 serves as a memory of recent inflammatory signaling (58, 63).

A20-binding inhibitor of NFκB activation 1(ABIN1; also known TNIP1) is a TNF-inducible binding partner of A20 (84–86). ABIN1 modulates A20-mediated inhibition of IKK-NFκB signaling by enhancing the de-ubiqutination of the IKK regulatory subunit, IKKγ/NEMO (84). The exact mechanism of ABIN1-mediated inhibition of IKK has yet to be elucidated. The observation that ABIN1 has a high affinity for polyubiquitin chains has informed some candidate mechanisms (87). One potential mechanism involves ABIN1 serving as an adaptor that brings A20 and its targets into close proximity (88). Another potential mechanism involves competition with the regulatory subunit of IKK, IKKγ/NEMO for polyubiquitin binding (88). Similar to the loss of A20, the loss of ABIN1 (*tnip1*^−/−^) may lead to embryonic lethality (89). *Tnip1*^−/−^ mice that reach adulthood develop autoimmune disorders spontaneously (87, 90). ABIN3 is another TNF-inducible binding partner of A20 (18, 91). The significance of ABIN3-mediated negative regulation of TLR-NFκB signaling has yet to be established and the mechanism has yet to be elucidated.

Monocyte chemotactic protein [MCP]-induced protein 1 (MCPIP1; also known as Regnase-1a or ZC3H12A) is a TNF-, IL1β-, and IL4-inducible deubiquitinase that negatively regulates NFκB activity (92–94). In the absence of MCPIP1, TLR-induced IKK phosphorylation, and NFκB nuclear translocation are enhanced as a result of elevated TRAF6 ubiquitination (93). The biological importance of MCPIP1 is highlighted by the fact that *Zc3h12*a^−/^^−^ mice develop lymphadenopathy, splenomegaly, growth retardation, and chronic autoimmunity and die prematurely (92, 93).

## NFκB-IκB Module

### IκB Synthesis

**Figure 5:**
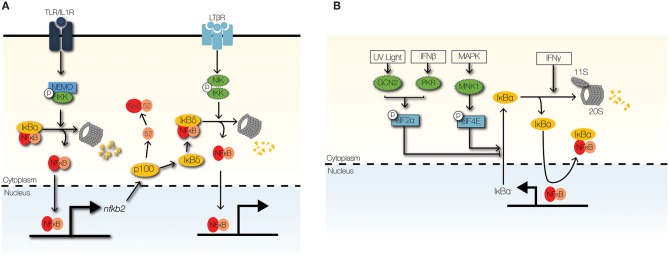
Signaling crosstalk at the IκB module. **(A)** The non-canonical NFκB signaling pathway can cross-regulate the canonical NFκB through NIK-IKK1-mediated degradation of IκBδ. High-molecular weight complexes of IκBδ trap RelA:p50 dimers in the cytoplasm to limit inflammatory NFκB activity. **(B)** Stimulus-responsive transcription initiation factors regulate the synthesis of IκBα. GCN2 and PKR phosphorylate eIF2α to inhibit IκBα synthesis in response to UV light and IFNβ, respectively. In contrast, phosphorylation of eIF4E by MNK1 stabilizes the IκBα mRNA. IFNγ promotes the proteolysis of IκBα/ε by upregulating the 11s cap of the immunoproteasome.

Regulation of IκBα synthesis via translational control of *nfkbia* mRNA, which encodes IκBα, can mediate cross-regulation of NFκB activity (Figure 5B). Type I IFNs, such as IFNβ, enhance TLR-NFκB signaling by repressing the translation of *nfkbia* (19). Further, stress responses to ultraviolet radiation (UV) and unfolded proteins (UPR) enhance NFκB activity through translation repression of *nfkbia* (95, 96). Translation of *nfkbia* is controlled by eukaryotic initiation factor (elF)2α and eIF4E [J. (97, 98)]. Translational repression of *nfkbia* by eIF2α depends on its phosphorylation by eIF2α kinases, such as PKR (interferon-induced, double-stranded RNA-activated protein kinase), PERK (pancreatic eIF2α kinase/RNA-dependent-protein-kinase-like endoplasmic-reticulum kinase), and GCN2 (general control non-derepressible-2) (96, 97, 99, 101). Whereas, PKR is activated by type I IFNs, GCN2, and PERK are activated by UV and UPR, respectively (100, 101).

IFNγ may also inhibit *nfkbia* translation and enhance NFκB activity by inhibiting the phosphorylation and activation of eIF4E (102). eIF4E-dependent inhibition of IκBα is controlled by MAPK and mammalian target of rapamycin (mTOR) pathways (98, 102). Interestingly, translation inhibition of IκBα significantly upregulates IFNβ production in response to double-stranded RNA stimulation (98). This observation hints at the possibility of positive feedback regulation of NFκB activity by type I IFNs. Currently, detailed investigations to examine this positive feedback regulation are lacking.

### IκB Degradation

Control of IκB degradation can mediate signaling crosstalk to NFκB (Figure 5B). IFNγ enhances NFκB activity by enhancing the degradation of free IκBα, which are unbound to NFκB dimers (19). Free IκBs have short half-lives (<10 min) and can be degraded independently of IKK activity and ubiquitination (99, 103); however, proteolysis of free IκBs is dependent on proteasomal degradation (99, 103). IFNγ enhances proteolysis of free IκBα by the immunoproteasome, which shares the 20S core of the 26S proteasome, but utilizes an 11S cap rather than a 19S cap (19, 104). IFNγ upregulates key components of the IκBα-associated 11S cap: PA28α and PA28β (19). Furthermore, pathological TNF signaling enhances NFκB activity by upregulating the degradation of IκBε by the immunoproteasome in a murine model of inflammatory bowel disease (105). TNF induces the expression PA28γ component of the immunoproteasome cap in colonic epithelial cells, which leads to severe colonic inflammation due to elevated NFκB activity (105).

### NFκB Trapping

Cytoplasmic trapping of RelA:p50 dimers by high-molecular weight IκB complexes (IκBsomes) permits multiple layers of inflammatory regulation (106, 107). It provides a gateway for crosstalk through developmental signals and provides a history of recent inflammatory signaling (Figure 5A). Members of the TNF receptor superfamily that transduce developmental signals, such as B-cell activator factor and lymphotoxin-β (LTβ), induce degradation of IκBδ, which is induced in response to inflammatory stimuli such as TLR ligands (108, 109). Although it is induced less rapidly than IκBα, IκBδ possesses a longer half-life and may function as a late brake on NFκB activity (110). Since IκBδ levels are invariant to canonical IKK-degradation, IκBδ functions as regulator of available NFκB dimers that can be activated by inflammatory stimuli (108). Finally, in the absence of IκBδ, priming with TNF or IL1β enhances NFκB signaling rather than inhibiting NFκB signaling (110).

## Concluding Remarks

Maintaining a delicate balance between effective host defense and deleterious inflammatory responses requires precise control of NFκB signaling (111). Multiple regulatory circuits have evolved to fine-tune NFκB-mediated inflammation through context-specific crosstalk (112). In this work, we have highlighted specific components of the NFκB signaling pathway for which crosstalk regulation is well-established. Despite decades of research, our current understanding of NFκB signaling remains insufficient to yield effective pharmacological targets (111, 113). Effective and specific pharmacological modulation of NFκB activity requires detailed, quantitative understanding of NFκB signaling dynamics (57). Furthermore, achieving cell-type and context-specific modulation of NFκB would be a panacea for many autoimmune and infectious diseases, as well as malignancies (112–114).

To dissect the dynamic regulation of NFκB signaling, quantitative approaches with single-cell resolution are required (115). By measuring the full distribution of signaling dynamics and gene expression in single cells, rather than simple averages, one can decipher cell-intrinsic properties from tissue-intrinsic properties (116–118). Such single-cell analyses may reveal strategies for targeting pathological cell populations with high specificity, which can mitigate adverse effects of pharmacological therapy (57, 113). Furthermore, with the aid of mathematical and computational modeling, one can conduct experiments *in silico* that may be prohibitive *in vitro* or *ex vivo* (57, 119, 120).

Finally, cross-regulatory pathways may fine-tune NFκB activity in a gene-specific manner. Many studies have identified the molecular components of gene-regulatory networks (GRNs) that control NFκB-dependent gene expression (15, 121). The regulatory mechanisms that define the topology of these GRNs include chromatin remodeling, transcription initiation and elongation, and post-transcriptional processing (15). They allow for combinatorial control by multiple factors and pathways, as well as cross-regulation (15). Further work will be required to delineate them in various physiological contexts.

## Author Contributions

AA conducted the literature review, prepared figures, and wrote the manuscript. AH provided supervision, outlined the scope, and edited the manuscript.

### Conflict of Interest Statement

The authors declare that the research was conducted in the absence of any commercial or financial relationships that could be construed as a potential conflict of interest.
